# Phase Formation of Multielement Nanoparticles from Immiscible Elements in Electrically Exploding Joint-Twisted Wires

**DOI:** 10.3390/nano16010040

**Published:** 2025-12-28

**Authors:** Kun Wang, Si’ao Zhang, Jiacheng Wang, Zhiyuan Li, Weikang Zhou

**Affiliations:** 1 State Key Laboratory of Smart Power Distribution Equipment and System, Hebei University of Technology, Tianjin 300130, China; 13230272669@163.com (J.W.); z445435323@163.com (W.Z.); 2 China Nuclear Power Engineering Co., Ltd. Hebei Branch, Shijiazhuang 050000, China; 15128219953@163.com

**Keywords:** exploding joint-twisted wires, multielement nanoparticles, phase state

## Abstract

This paper presents the experimental and computational results characterizing the phase formation of multielement nanoparticles synthesized by the electrically exploding joint-twisted wires. The joint-twisted wires with different element compositions are exploded to investigate the influence of immiscible elements on the phase states of the multielement nanoparticles. The element contents of the multielement nanoparticles deviate from their initial element proportions of the joint-twisted wires due to the non-synchronous exploding process. The silver element enriches the nanoparticle surface, while aluminum, iron, cobalt, and nickel elements show a homogeneous distribution within the nanoparticle. The phase segregation can be adjusted by changing the initial proportion of the silver element in the joint-twisted wires. The decrease in the proportion of silver in joint-twisted wires promotes the homogeneity of silver in the multielement nanoparticles with the phase structure transition from the BCC phase to the FCC phase. A molecular dynamics simulation suggests that both higher initial temperature and more uniform initial mixing conditions facilitate the homogeneous merging of all elements. This study helps with gaining a deep understanding of the phase formation of multielement nanoparticles synthesized by the electrically exploding joint-twisted wires.

## 1. Introduction

Nanoparticles are of great interest in catalysts [1], magneto-functional nanomaterials [2], and biomedical applications [3]. Diverse pure elements blended in multielement nanoparticles (ME-NPs) induce multielement synergy, enhancing the physical and chemical properties of the nanoparticles [4]. The high-entropy alloy nanoparticles (HEA-NPs), which represent a class of ME-NPs containing more than four elements of equal stoichiometric ratios uniformly mixed into a single-phase solid-solution alloy, offer a huge material library with virtually unlimited compositional space [5]. The wide range of possible compositions poses an enormous challenge in the controlled incorporation of multiple elements into a nanoscale particle. The miscibility of elements in nanoparticles differs dramatically from that of their bulk counterparts [6]. The complete miscibility of elements at the nanoscale is usually achieved under extreme conditions. The carbothermal shock approach with heating and cooling rates up to 10^5^ K/s creates a breakthrough in the synthesization of HEA-NPs [7]. The general synthesis methods suggest that the growth of the ME-NPs is controlled by the heating and cooling rates [8,9]. Intense heating of the metal target followed by a rapid quenching treatment can suppress the occurrence of phase segregation.

The electrically exploding wires have been commercialized in the mass production of the elementary nanoparticles. It has the advantages of great energy utilization efficiency, controllable preparation in size distribution, and a high degree of purity [10]. The pulsed current generates a temperature rise rate of approximately 10^9^ K/s, driving the metal wires to experience a phase transition from a solid state to a gaseous state, even to a plasma state. The exploding products evolve into nanoparticles under the cooling action of the ambient inert gases through the processes of nucleation, coagulation, and surface growth [11]. Recently, the joint electrically exploding wires successfully synthesized the AlCrFeCuNi nanoparticles [12], which manifests promising potential for the continuous synthesis of various ME-NPs. The high cooling rate of the dispersed system in the non-equilibrium exploding process mixes metallic components irrespective of electrical conductivity, melting temperature, and the heat capacity of individual elements [13]. A great deal of research aims to reveal the structures and phase compositions of the ME-NPs formed in the joint electrically exploding wires [14,15,16]. The wide range of possible compositions and atomic arrangements adds layers of complexity in the phase states of the ME-NPs. For instance, in terms of relatively simple binary alloy nanoparticles, two immiscible metals can fuse into homogeneous mixing, core–shell, and Janus-type structures [17]. It is suggested that atomic clusters originating from metals of low density, low melting temperature, and low surface energy grow on the surface of a nanoparticle to form a core–shell structure, while the deformation of the interface caused by different crystalline structures provides additional energy to convert the structure from the core–shell type to the Janus type. The high mixing entropy, increasing the disorder degree of the alloy system, tends to form a simple BCC or FCC solid solution phase. The metal wire is exploded into liquid clusters and vapor–plasma mixtures [18]. The exploding products of several elements mixed uniformly in an inert environment facilitate the homogeneous synthesization of the ME-NPs. However, the ME-NPs suffer from phase segregation caused by the immiscibility of mixing elements [19]. The merging of different elements is a key issue in the phase formation of ME-NPs in electrically exploding wires.

In this paper, the experimental and computational results characterizing the phase formation of ME-NPs synthesized by the electrically exploding joint-twisted wires are presented. Three samples of joint-twisted wires with different element compositions are exploded to elucidate the influence of the immiscible element on the phase formation of ME-NPs. The rest of this paper is organized as follows: Section 2 describes the experiment setup. Section 3 is the results and discussion. Section 4 gives the conclusion.

## 2. Experimental Setup

**Figure 1:**
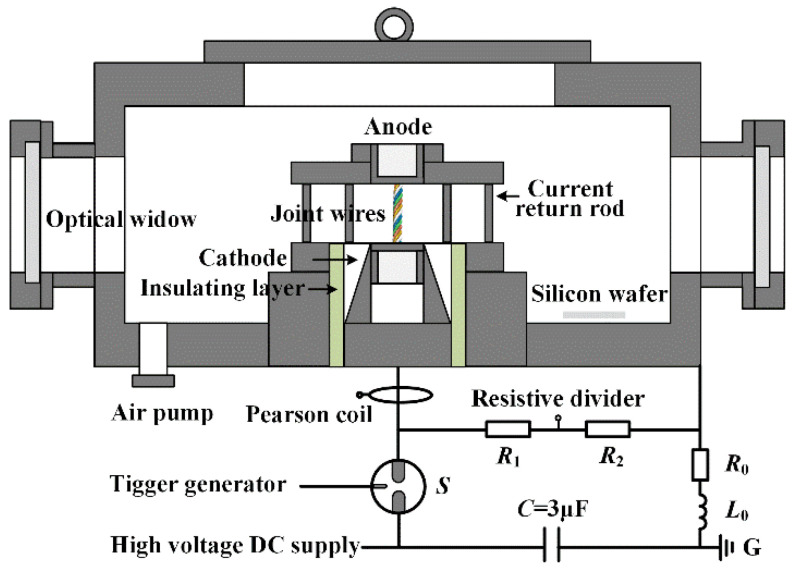
The schematic diagram of the experimental setup.

The experiments for synthesization of the ME-NPs by the electrically exploding wires are performed using a compact pulsed power generator. The wires of different elements are wound by a small motor to form a joint-twisted structure. The diagram of the experimental setup is shown in Figure 1. The energy storage capacitor *C* is charged by a high-voltage direct current power supply. The energy storage capacitor discharges through a three-electrode gas switch *S* controlled by a trigger generator. The joint-twisted wires’ load is fixed by a coaxial target unit in a vacuum chamber. The distance between the electrodes is 2.5 cm. The chamber is vacuumized and then filled with argon gas to a pressure of 0.1 MPa. The intrinsic resistance, *R*_0_ = 76 mΩ, and inductance, *L*_0_ = 1.74 μH, of the discharge circuit are estimated from the short circuit experiments. The current flowing through the wire load is measured by a Pearson coil (Model 101, Pearson Electronics, Inc., Palo Alto, CA, USA). The voltage across the electrodes is measured by a resistive divider composed of a high-voltage resistor *R*_1_ and a low-voltage resistor *R*_2_. The ratio of the resistive divider is 1 mV/V. A Tektronix MDO3104 oscilloscope (Tektronix Inc., Beaverton, OR, USA) is used to record the electrical signals.

The metal elements of silver (Ag), aluminum (Al), iron (Fe), cobalt (Co), and nickel (Ni) are employed in the experiments. Three samples of the joint-twisted wires are exploded to synthesize the ME-NPs. The detailed configurations of the joint-twisted wire loads are listed in Table 1. The variable *d* with subscripts of different elements represents the wire diameter of corresponding element. *E_s_* is the energy required to fully vaporize the joint-twisted wires. The synthesized nanoparticles diffuse from the explosion center to the peripheral space. Finally, the nanoparticles settle down at the bottom of the vacuum chamber. Thus, a silicon wafer is placed on the bottom of the vacuum chamber to collect the ME-NPs.

The nanoparticles are tested in the materials analysis center. The microscopic morphology of the nanoparticles is analyzed by the field emission transmission electron microscope (TEM, FEI Talos F200S, Thermo Fisher Scientific Inc., Waltham, MA, USA). Energy-dispersive X-ray spectroscopy (EDX) is used to detect the elemental distribution within the nanoparticles. The X-ray diffraction patterns are acquired by a Cu Kα radiation (*λ* = 0.15406 nm) X-ray diffractometer (XRD, Rigaku SmartLab 9 kW, Rigaku corporation, Akishima, Japan). The phase analysis of the nanoparticles is conducted using MDI Jade software (Version 6.5) based on the PDF-2 diffraction database.

## 3. Results and Discussion

**Figure 2:**
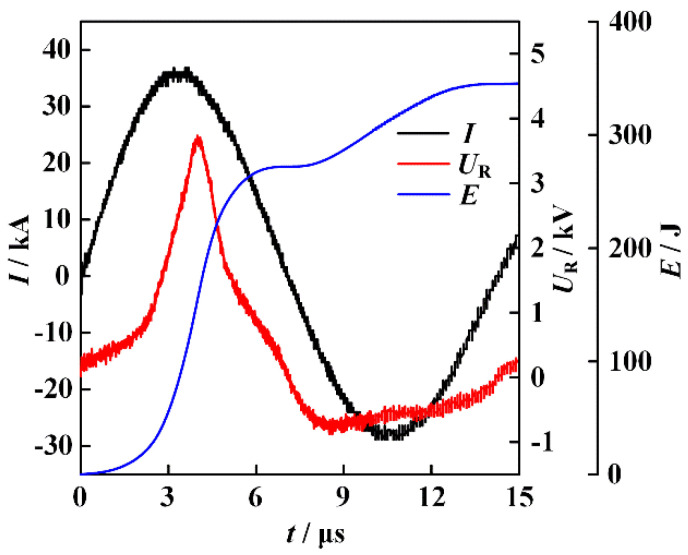
The typical waveforms of current, resistive voltage, and energy deposition for the electrically exploding joint-twisted wires (sample 1:150 μm-diameter Ag wire, 300 μm-diameter Al wire, 300 μm-diameter Fe wire, 300 μm-diameter Co wire, and 200 μm-diameter Ni wire).

The atomic percentage of the elements involved in the formation of ME-NPs can be conveniently adjusted by changing the diameter of the counterpart wires. In the experiments, the energy storage capacitor is charged to 30 kV. The typical waveforms of current and resistive voltage for the electrically exploding joint-twisted wires of sample 1 are shown in Figure 2. The joint-twisted wire load is composed of 150 μm-diameter Ag wire, 300 μm-diameter Al wire, 300 μm-diameter Fe wire, 300 μm-diameter Co wire, and 200 μm-diameter Ni wire.

The resistive voltage is reconstructed by subtracting the inductive part from the measured data. The pulsed current with 36.8 kA in magnitude and 10 A/ns in rise rate drives the joint-twisted wires to experience a severe phase transition. The voltage peaks at the instant *t*_0_ = 3.8 μs, at which the voltage breakdown occurs. An energy of 153 J is deposited into the joint-twisted wires during the resistive stage. The amount of energy deposition at the instant of voltage breakdown is approximately 45% of the energy required to fully vaporize the entire wire load. Although the energy deposition is suppressed by the formation of the corona plasma channel, the dense core further absorbs energy from the residual current and the thermal exchange during the process of voltage collapse. It is suggested that the energy deposition should be calculated up to the moment when the wire resistance decreases to half of its maximum value [20]. The resistance decreases to half of its maximum value at the instant *t*_1_ = 5.6 μs. At this moment, the total energy deposited into the joint-twisted wires’ load is 259 J, transforming 77% of the wire load into a gaseous state. Applying shorter wires can further enhance the energy deposition [21]. It can be seen from Figure 2 that the asynchronization in voltage breakdown is indistinguishable. The fast expansion of the conductive corona plasmas renders the voltage breakdown of individual wires negligible in the voltage waveform.

**Figure 3:**
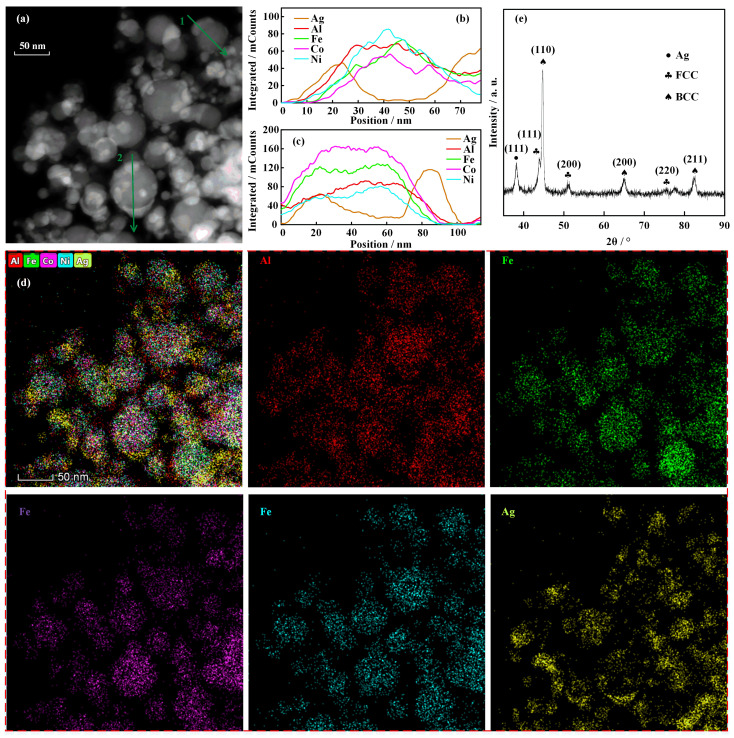
The morphology and elemental analysis data of the AgAlFeCoNi nanoparticles synthesized by the electrically exploding sample 1: (**a**) TEM image; (**b**,**c**) element distribution by line scanning along the green arrows in (**a**); (**d**) elemental mappings; (**e**) XRD pattern.

The morphology and elemental analysis data of the AgAlFeCoNi nanoparticles synthesized by electrically exploding sample 1 are shown in Figure 3. One can see from the TEM image in Figure 3a that the nanoparticles produced by the electrically exploding joint-twisted wires are generally spherical in shape. The processes of nucleation, coagulation, and surface growth dominate the growth of nanoparticles, resulting in a lognormal distribution in particle size. The average diameter of the nanoparticles is 20.5 nm. The nanoparticles of Janus-type structure are observed in the TEM image, indicating that certain elements concentrate on the surface of the nanoparticles. Two nanoparticles marked by green arrow 1 and arrow 2 exhibit an outstanding characteristic of phase segregation. The element distributions along the green arrows for nanoparticles 1 and 2 are shown in Figure 3b,c, respectively. The curves for element distribution show that Al, Fe, Co, and Ni elements are mixed uniformly. However, the Ag element concentrates on the surface of the nanoparticles. The elemental mappings in Figure 3d also clearly demonstrate that Al, Fe, Co, and Ni elements distribute uniformly within a nanoparticle volume. This is a benefit of their good miscibility. For a two-phase system, the mixing enthalpies between the Ag element and Al, Fe, Co, and Ni elements are positive, making it difficult to form an alloy. The positive mixing enthalpy and low cohesive energy are responsible for the segregation of the Ag element. Furthermore, the large atomic radius and low surface energy prompt Ag element enrichment on the nanoparticle surface.

The data of XRD analysis for the AgAlFeCoNi nanoparticles are shown in Figure 3e. It can be seen from the XRD patterns that the crystalline structures of FCC and BCC lattices are observed in nanoparticles. The lattice parameters of the FCC lattice and BCC lattice are 4.27 Å and 3.81 Å, respectively. A small amount of the FCC phase with a lattice parameter of 4.0816 Å is observed in the nanoparticles. The lattice parameters of equilibrium states for Al and Ag are 4.049 Å and 4.086 Å [22]. Thus, it can be identified as an Ag-rich phase. The low cohesion energy for the Ag element makes it easy to combine Ag atoms to form the Ag-rich phase during solidification. The weight percentages of the FCC phase and BCC phase are 68.6% and 18.9%, respectively. The Ag-rich phase accounts for the remainder of the weight percentage.

The metal wires accumulate sufficient energy under the intense Joule heating of the pulsed current to transit into a two-phase state. The exploding products are heated to a temperature of several electron volts. Subsequently, the temperature of exploding products drops gradually. Theoretical and experimental studies have shown that the exploding products are a mixture of condensed-phase clusters and partially ionized plasma [23]. The vapor atoms collide at a proper supersaturated state to form stable liquid nuclei. The deposition and vaporization of vapor atoms occur simultaneously on the surface of the liquid nuclei. Liquid clusters of different elements coalesce into droplets at high temperatures. Finally, nanoparticles of smaller size grow into larger nanoparticles through collision and coagulation processes. The ambient gas cools down the clusters by three-body collisions. The nanodroplets solidify when the temperature drops to the melting point. It is evident that the crystalline phases of the ME-NPs can be controlled over the thermodynamic factors and the solid-state diffusion process.

**Figure 4:**
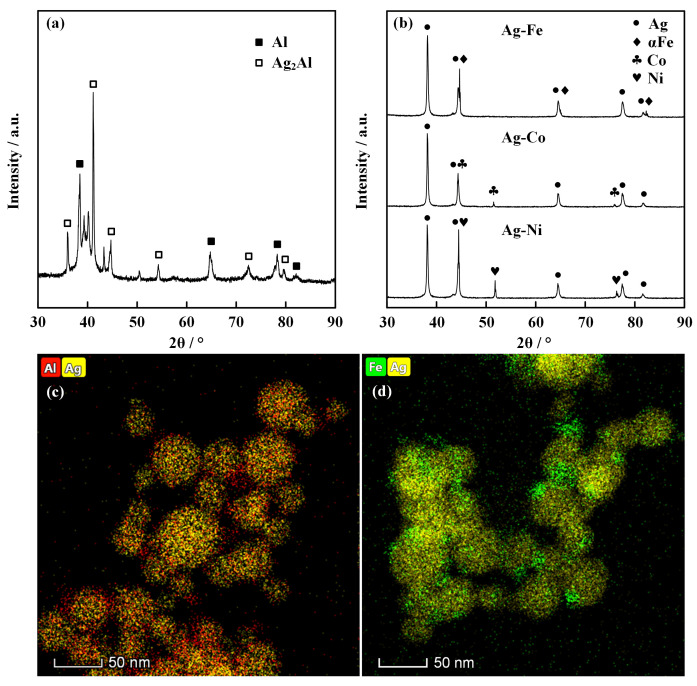
The XRD data and EDS patterns of the binary nanoparticles: (**a**) XRD data of Ag-Al nanoparticles; (**b**) XRD data of Ag-Fe, Ag-Co, and Ag-Ni nanoparticles; (**c**) and (**d**) are EDS patterns of Ag-Al and Ag-Fe binary nanoparticles, respectively.

The experimental results of the electrically exploding Sample 1 reveal the occurrence of Ag segregation in the ME-NPs. Since the ME-NPs originate from a mixture of vapor atoms and liquid clusters, the mixing enthalpy plays a crucial role in determining the structural characteristics during the alloy solidification. The significant difference in the atomic size and crystal parameters of metals leads to immiscibility or nearly complete insolubility between elements [24,25]. The high binary mixing enthalpy between Ag and Al, Fe, Co, and Ni promotes a pronounced phase separation [26]. The bimetallic nanoparticles of Ag-Al, Ag-Fe, Ag-Co, and Ag-Ni are synthesized by the electrically exploding twin wires. The XRD data and EDS patterns of the binary nanoparticles are shown in Figure 4. Figure 4a is the XRD data of Ag-Al binary nanoparticles. One can see that the Ag_2_Al and Al are observable in Ag-Al nanoparticles. Figure 4b demonstrates the XRD data for Ag-Fe, Ag-Co, and Ag-Ni binary nanoparticles. In contrast to the Ag-Al nanoparticles, the intermetallic phase is not detected in Ag-Fe, Ag-Co, and Ag-Ni nanoparticles. The Ag-Fe, Ag-Co, and Ag-Ni nanoparticles contain the initial metallic phases.

The EDS pattern of the Ag-Al nanoparticles, as shown in Figure 4c, exhibits a relatively homogeneous distribution of the Ag and Al elements, indicating that limited mixing might occur between immiscible metals. Due to the difference in lattice parameters and surface energy, the combination of different metals tends to undergo phase separation in accordance with the binary equilibrium phase diagram. Thus, in the circumstance of Ag-Fe nanoparticles, as shown in Figure 4d, the binary nanoparticles primarily form a Janus-type structure with minor fractions of uniformly mixed phases. According to the Hume-Rothery rules, Fe, Co, and Ni are similar to one another in terms of atomic radius, electronegativity, valence electron concentration, and electron configuration, all of which differ considerably from those of Ag. On the other hand, Fe, Co, and Ni exhibit similarly high positive binary mixing enthalpies with Ag, which induces mutual repulsion between these transition metal atoms and Ag atoms. Consequently, the system tends to lower its free energy via phase separation [27]. The similarity of properties for Fe, Co, and Ni elements allows the Ag-Fe, Ag-Co, and Ag-Ni nanoparticles to retain the intermetallic phase and form a Janus-type structure. In contrast, the Ag-Al binary system has a negative mixing enthalpy. The Al and Ag possess an FCC crystal structure. The small mismatch in atomic radius and significant difference in electronegativity are in favor of the formation of ordered intermetallic compounds rather than solid solutions for Ag-Al nanoparticles. The positive mixing enthalpy of blending the Ag element into the other four elements to form the quinary alloys, as well as the poor affinity, account for the phase separation observed in the ME-NPs.

**Figure 5:**
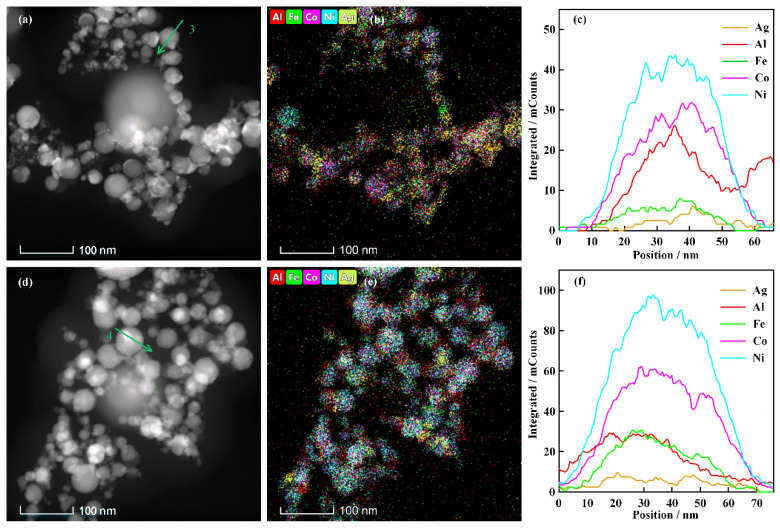
The morphology and element analysis data of ME-NPs synthesized by the electrically exploding Sample 2 and Sample 3: (**a**–**c**) are the TEM image, elemental mapping, and element distribution by line scanning for Sample 2; (**d**–**f**) are the TEM image, elemental mapping, and element distribution by line scanning for Sample 3.

The high-entropy alloys are characterized by random occupation of lattice sites by atoms of each constituent element. They tend to form and maintain solid solutions during alloying. The elemental composition significantly influences the atomic arrangement and lattice parameter of high-entropy alloys [28]. The morphological image and element analysis data for the electrically exploding Sample 2 and Sample 3 are shown in Figure 5. The elemental line scanning data in Figure 5c and Figure 5f is performed along the green arrows in Figure 5a and Figure 5d, respectively. The experimental data from Sample 2 demonstrate that elements of Al, Fe, Co, and Ni distribute uniformly within the nanoparticles, while the Ag element forms the element segregation. The degree of element segregation is less severe than that of Sample 1. The nanoparticles synthesized by the electrically exploding Sample 3 exhibit a homogeneous distribution of all five elements. The contents of Ag and Al elements in nanoparticles are higher than their initial proportions in joint-twisted wires, whereas the contents of Fe, Co, and Ni are lower than their initial proportions. The non-synchronous exploding process might be responsible for the deviation of element contents in nanoparticles from their initial proportions in joint-twisted wires. The explosion time can be estimated by *τ*_ex_~*h*/*j*^2^ [29], in which *h* is the specific current action integral for heating the wire from the room temperature to the explosion point, and *j* is the current density. It is estimated that the explosion times for Ag and Al wires are shorter than those of Fe, Co, and Ni wires, with the assumption that the redistribution of current density is primarily determined by the resistivity at the initial stage of the discharge, resulting in a non-synchronous exploding process of individual wires. The Ag and Al wires reach their melting points earlier to eject more submicron droplets to participate in the formation of ME-NPs. The experimental measurement demonstrates that the non-synchronous exploding process exerts no influence on the phase separation of Ag in the nanoparticles. Conversely, the reduced presence of Fe, Co, and Ni elements in the nanoparticles suggests that higher proportions of Fe, Co, and Ni elements are retained in micron-scale droplets.

**Figure 6:**
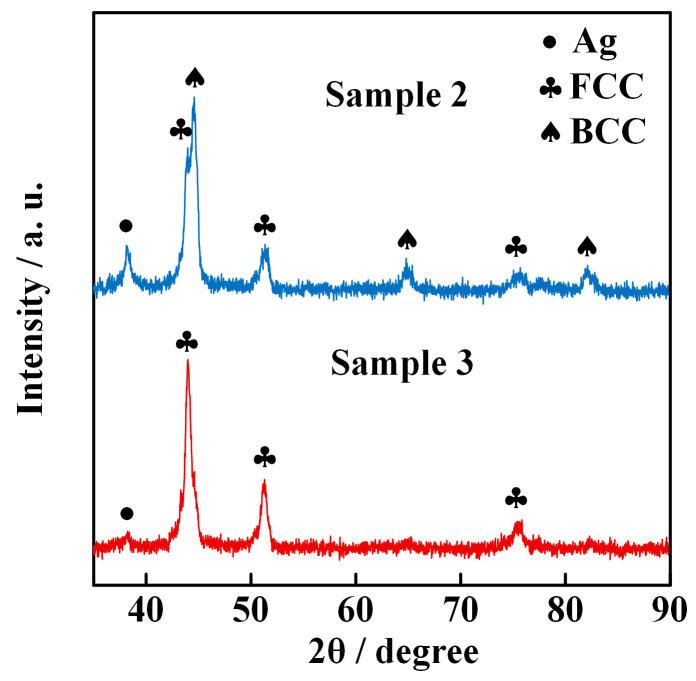
XRD data of ME-NPs synthesized by the electrically exploding Sample 2 and Sample 3.

The XRD data of ME-NPs synthesized by the electrically exploding Sample 2 and Sample 3 are shown in Figure 6. The ME-NPs from Sample 2 contain the FCC phase, BCC phase, and Ag phase with corresponding mass fractions of 40.8%, 52.5%, and 6.7%, respectively. In contrast, the ME-NPs from Sample 3 comprise the FCC solid solution with 97.2% in mass fraction and Ag with 2.8% in mass fraction. The phase compositions of the ME-NPs change with the proportion of elements in different configurations of joint-twisted wires. The Al element introduces lattice strain due to its large atomic radius, promoting a transition from an FCC structure to a BCC structure in FeCoNiAl-based high-entropy alloys [30]. The Ag element also possesses a larger atomic radius than that of Fe, Co, and Ni elements. Thus, mixing the Ag element contributes strain energy to overcome the energy barrier between the FCC and BCC structures. The reduction in the total contents of Al and Ag elements in the AgAlFeCoNi nanoparticles drives the change in crystal structure. As the Ag content in the wire load decreases, the Ag element gradually diminishes and eventually almost disappears in the nanoparticles. Thus, the electrically exploding Sample 3 tends to form nanoparticles of a single-phase FCC solid solution.

The surface element contents of AgAlFeCoNi nanoparticles are listed in Table 2. Several critical characterization parameters are calculated based on the surface element contents. Δ*S*_mix_ and Δ*H*_mix_ are the mixing entropy and mixing enthalpy, respectively. *δ* is the atomic size difference. VEC is the abbreviation of valence electron concentration, which represents the average number of valence electrons per atom in the ME-NPs. Although the Δ*S*_mix_ of nanoparticles from Sample 1 satisfies the criterion of HEA-NPs, the nanoparticles exhibit the most pronounced Ag segregation phenomenon. The nanoparticles from Samples 2 and 3 are classified as medium-entropy alloys from the view of mixing entropy. The negative Δ*H*_mix_ and small δ of ME-NPs facilitate the bonding of atoms. The positive binary mixing enthalpies between Ag and Ni (15 kJ·mol^−1^), Co (19 kJ·mol^−1^), and Fe (28 kJ·mol^−1^) result in the highest mixing enthalpy for Sample 1. The decrease in Ag content and increase in Ni content promote a more homogeneous elemental distribution in the ME-NPs. The stability of solid solutions in ME-NPs arises from a thermodynamic balance between limited Δ*H*_mix_ and high Δ*S*_mix_ during the multielement alloying [31].

The parameter of VEC is used to predict the stable solid solution structure. If the VEC exceeds 8.0, the high-entropy alloy is in the FCC phase. Part of the phase state transforms to the BCC phase with the decrease in VEC until a threshold of 6.87, below which only the BCC phase exists [32]. Higher VEC reduces the space among atoms in the matrix, resulting in a higher atomic density, which is in favor of the formation of the FCC phase [33]. The phase composition predicted by the VEC parameters is consistent with the experimental XRD results. The nanoparticles from Sample 1 and Sample 2 form both BCC and FCC phases with intermediate VEC values. The alloy is more prone to precipitate an Ag-rich segregation phase with larger lattice parameters. The increased Ni content in Sample 3 plays a key role in stabilizing the FCC phase [34]. The rearrangement of atoms within the crystal lattice leads to the formation of a single-phase FCC structure, which inhibits the occurrence of the Ag segregation phase. Therefore, the ME-NPs with homogeneous elemental distribution are obtained in the electrically exploding Sample 3.

The exploding products are a mixture of partially ionized plasma and liquid metal clusters. The mixing of liquid-state nanoclusters serves as a crucial mechanism for the formation of alloy nanoparticles in the electrically exploding wires [35]. Two physical scenarios are considered to account for the formation of HEA-NPs. The first physical scenario involves the process of condensed-phase clusters undergoing collision, coalescence, and condensation of individual single-element clusters. The individual nanoclusters are electrically neutral spheres with a diameter ranging from 3 to 5 nm, randomly placed in the computational domain filled with argon gas atoms. The second physical scenario describes the gaseous atoms of different elements uniformly mixed to form HEA-NPs through the nucleation, surface growth, and condensation processes. The Ag, Al, Fe, Co, and Ni atoms are randomly distributed in a spherical nanoparticle with an equal atomic ratio to simulate homogeneous vapor mixtures. The total atomic number is 43,227. A molecular dynamics simulation is performed to investigate the formation of HEA-NPs in an argon gas environment. Since the relaxation time of the exploding plasma is significantly shorter than the time required for the exploding products to cool down to a point of triggering the nucleation process [36,37], it is assumed that nanoclusters of different elements are at the same temperature in the ambient gas at the onset of the nanoparticle growth. The interatomic interactions of metal atoms are described by a semi-empirical potential based on the embedded atom method [38]. This alloy embedded atom method potential database of sufficient generality has enabled alloy potentials for more than a dozen metals [39]. The initial temperature of the nanoclusters is determined by assigning random velocities following the Gaussian distribution. The interaction between metal atoms and argon molecules is described by the Lennard-Jones potential function. The time step for the molecular dynamics simulation is set to 1 fs. The ambient gas temperature and pressure are set to 300 K and 100 kPa, respectively. The computation terminates when the clusters evolve into a stable structure.

**Figure 7:**
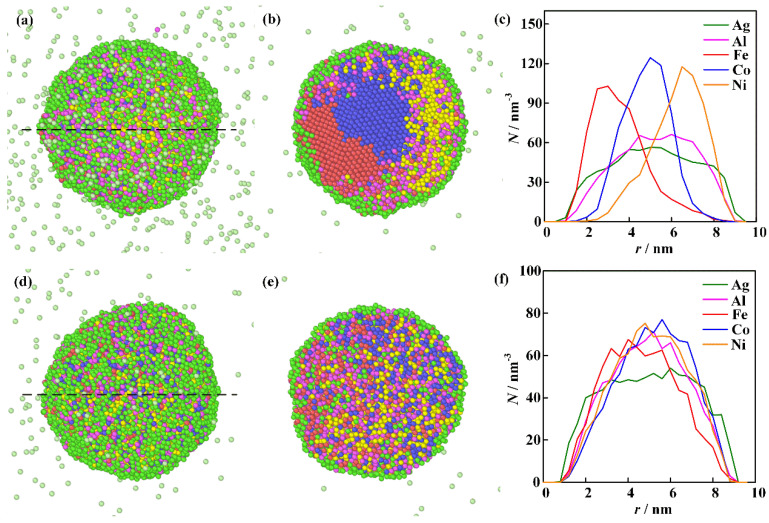
The computational results for the merging of individual single-element nanoclusters at different initial temperatures: (**a**–**c**) are the integral shape, cross-section morphology, and elemental distribution along the black dashed line in (**a**) at a temperature of 2000 K; (**d**–**f**) are the integral shape, cross-section morphology, and elemental distribution along the black dashed line in (**d**) at a temperature of 3000 K.

The computational results for the merging of individual single-element nanoclusters described in the first physical scenario at different initial temperatures are shown in Figure 7. The green, purple, red, blue, and yellow spheres represent Ag, Al, Fe, Co, and Ni atoms, respectively. Figure 7a–c are results calculated under the initial temperature of 2000 K. During the process of the nanocluster aggregation, the temperature of the clusters is determined by the interaction between metallic atoms and the argon gas, as well as the released thermal energy converted from the atomic binding energy [40]. One can see that the surface of the nanoparticle is primarily covered by Ag atoms. The elemental distribution curve along the black dashed line reveals that the phase separation within the nanoparticle is outstanding. The energy released during the cluster coalescence provides insufficient driving force for the homogeneous elemental mixing. Thus, the elements of high melting points, such as Fe, Co, and Ni, are distributed unevenly within the nanoparticle. The Ag element with low melting temperature and surface energy moves toward the nanoparticle surface. Increasing the initial temperature enhances the thermal motion of atoms. The computational results under the initial temperature of 3000 K are presented in Figure 7d–f. One can see that the initial temperature exerts a significant influence on the mixing behavior of the clusters. Although the Ag segregation phenomenon is still observed on the surface of the nanoparticle, the homogeneity of Al, Fe, Co, and Ni atoms is greatly improved.

**Figure 8:**
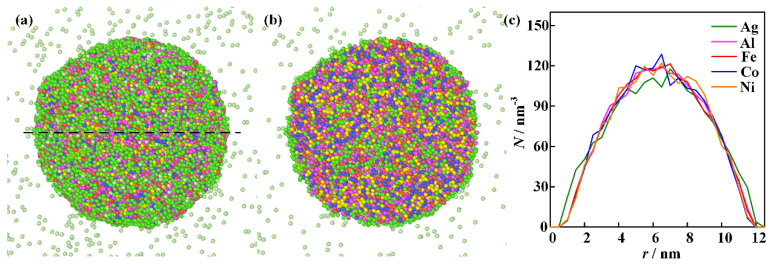
The computational results for the merging of vapor mixture: (**a**–**c**) are integral shape, cross-section morphology, and elemental distribution along the black dashed line.

The computational results for the nanoparticle formed from the homogeneous vapor mixtures described in the second physical scenario are shown in Figure 8. The initial temperature of the simulation is 2000 K. The driving force for atomic thermal motion originates from the tendency of the high-temperature mixture to reduce its Gibbs free energy [41]. The formation of a nanoparticle starts from a homogeneous mixing condition. The elements of Al, Fe, Co, and Ni are uniformly distributed within the nanoparticle, while most of the Ag atoms are migrated to the surface of the nanoparticle. The computational results reproduce the phase states of the ME-NPs characterized by the uniform distribution of Al, Fe, Co, and Ni elements with surface segregation of Ag atoms. The physical pictures align well with experimental measurements. The homogeneity of the elements increases with the decrease of Ag content. However, the solid solubility of immiscible elements in high-entropy alloy nanoparticles is quite limited.

**Figure 9:**
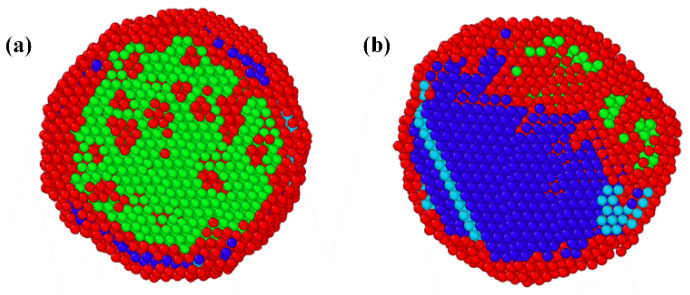
The cross-section of crystal structures after solidification of the clusters: (**a**) cluster 1; (**b**) cluster 2 (dark blue—FCC phase; light blue—HCP phase; green—BCC phase; red—unknown phase).

The molecular dynamics simulation is further developed to determine the crystal structure of AgAlFeCoNi nanoparticles. The cluster is in FCC configuration, containing 9045 randomly distributed atoms. The common neighbor analysis is performed to identify the phase structures. The simulation is conducted under the NVT ensemble. The nanoclusters are first stabilized at 1400 K to preserve the fundamental structure. Subsequently, the cluster is cooled down to the room temperature at a controlled rate of 1 × 10^10^ K/s. Clusters with different atomic compositions are considered in the computation. The atomic composition for cluster 1 is Ag 20%, Al 20%, Fe 20%, Co 20%, and Ni 20%. The atomic composition for cluster 2 is Ag 16%, Al 16%, Fe 24%, Co 20%, and Ni 24%. The crystal structures after solidification of the clusters are shown in Figure 9. The common neighbor analysis demonstrates that both nanoparticles contain FCC, BCC, HCP, and unidentified phases. The BCC phase is the dominating phase in cluster 1, while cluster 2 is predominantly the FCC phase with minor BCC content. The HCP phase arises from stacking faults and twin structure in the lattice of the FCC crystal structures [42]. The unidentified atoms on the nanoparticle surface constitute an amorphous phase characterized by disordered atomic arrangements. The high cooling rate, which gives insufficient time to rearrange and create a crystalline phase, is responsible for the unidentified atoms formed within the nanoparticle.

## 4. Conclusions

This paper reports the synthesization of ME-NPs in the electrically exploding joint-twisted wires containing Ag, Al, Fe, Co, and Ni elements. The pure metallic phase segregation is observed in ME-NPs containing immiscible metallic elements. The experimental data of the ME-NPs derived from electrically exploding Sample 1 demonstrate that the weight percentages of the FCC phase, BCC phase, and Ag-rich phase are 68.6%, 18.9%, and 12.5%, respectively. The ME-NPs from Sample 3 comprise the FCC solid solution with 97.2% in weight percentage and Ag with 2.8% in weight percentage. An essentially well-mixed single-phase FCC solid solution of ME-NPs is prepared by adjusting the initial element configurations. These experiments shed light on the phase formation mechanism of ME-NPs containing immiscible metallic elements. High mixing entropy promotes the formation of a solid solution phase. However, the enthalpy contribution to the total Gibbs free energy dominates over the entropy contribution when immiscible elements are involved in the formation of nanoparticles. The occurrence of elemental segregation is closely related to the mixing enthalpy and VEC. Specifically, metals with a positive mixing enthalpy and a moderate VEC are immiscible in the alloy. During the solidification, the behavior of element segregation is related to the properties of elements, encompassing melting point, density, and surface energy. Metals with low melting points, density, and surface energy are easily distributed on the surface of a nanoparticle. The molecular dynamics method is employed to investigate the formation mechanism of ME-NPs in electrically exploding wires. The results demonstrate that higher initial temperature promotes more homogeneous elemental distribution. Ag atoms consistently exhibit a tendency for surface segregation. The crystal structure of AgAlFeCoNi nanoparticles is analyzed, and it is found that the nanoparticles primarily consist of FCC phase, BCC phase, and a small amount of HCP phase. The amorphous layer is observed on the nanoparticle surface. These findings provide a rationale for the preparation of ME-NPs with a uniform structure using the electrically exploding wire method.

## Figures and Tables

**Table 1:** The detailed configurations of the joint-twisted wires.

Sample	*d*_Ag_, μm	*d*_Al_, μm	*d*_Fe_, μm	*d*_Co_, μm	*d*_Ni_, μm	*E*_s_, J
1	150	300	300	300	200	338
2	100	300	200	300	300	337
3	50	200	200	300	300	301

**Table 2:** The surface element content of AgAlFeCoNi nanoparticles.

Sample	Ag at.%	Al at.%	Fe at.%	Co at.%	Ni at.%	Δ*S*_mix_ J·mol^−1^·K^−1^	Δ*H*_mix_ kJ·mol^−1^	*δ* %	VEC
1	13.41	31.25	20.61	21.68	13.05	12.93	−6.28	1.25	7.32
2	5.9	32.06	8.58	23.17	30.29	12.00	−13.31	1.16	7.41
3	1.83	17.79	13.26	32.4	34.72	11.48	−10.34	0.74	8.18

## Data Availability

The original contributions presented in this study are included in the article. Further inquiries can be directed to the corresponding author.

## References

[1] Xie P.F., Yao Y.G., Huang Z.N., Liu Z.Y., Zhang J.L., Li T.Y., Wang G.F., Shahbazian-Yassar R., Hu L.B., Wang C. (2019). Highly efficient decomposition of ammonia using high-entropy alloy catalysts. Nat. Commun..

[2] Manjunatha K., Chiu H.H., Ho M.K., Hsu T.E., Yu S.L., Chin C.E., Cheng C.L., Carvalho de Oliveira M., Longo E., Ribeiro R.A.P. (2023). Mg-Doped CoCr_2_O_4_ nanoparticles: Implications for magnetic memory and magnetocaloric effect. ACS Appl. Nano Mater..

[3] Akinola P.O., Lateef A., Asafa T.B., Beukes L.S., Abbas S.H., Irshad H.M. (2022). Phytofabrication of titanium-silver alloy nanoparticles (Ti-AgNPs) by Cola nitida for biomedical and catalytic applications. Inorg. Chem. Commun..

[4] Liao Y.J., Li Y.X., Zhao R.Z., Zhang J., Zhao L.Z., Ji L.Z., Zhang Z.Y., Liu X.L., Qin G.W., Zhang X.F. (2022). High-entropy-alloy nanoparticles with 21 ultra-mixed elements for efficient photothermal conversion. Natl. Sci. Rev..

[5] Yao Y.G., Liu Z.Y., Xie P.F., Huang Z.N., Li T.Y., Morris D., Finfrock Z., Zhou J.H., Jiao M.L., Gao J.L. (2020). Computationally aided, entropy-driven synthesis of highly efficient and durable multi-elemental alloy catalysts. Sci. Adv..

[6] Chen P.C., Gao M.Y., McCandler C.A., Song C.Y., Jin J.B., Yang Y., Maulana A.L., Persson K.A., Yang P.D. (2024). Complete miscibility of immiscible elements at the nanometre scale. Nat. Nanotechnol..

[7] Yao Y.G., Huang Z.N., Xie P.F., Lacey S.D., Jacob R.J., Xie H., Chen F.J., Nie A., Pu T.C., Rehwoldt M. (2018). Carbothermal shock synthesis of high-entropy-alloy nanoparticles. Science.

[8] Wang B., Wang C., Yu X.W., Cao Y., Gao L.F., Wu C.P., Yao Y.F., Lin Z.Q., Zou Z.G. (2022). General synthesis of high-entropy alloy and ceramic nanoparticles in nanoseconds. Nat. Synth..

[9] Yao Y.G., Dong Q., Brozena A., Luo J., Miao J.W., Chi M.F., Wang C., Kevrekidis Y., Ren Z.Y.J., Greeley J. (2022). High-entropy nanoparticles: Synthesis-structure-property relationships and data-driven discovery. Science.

[10] Jamkhande P.G., Ghule N.W., Bamer A.H., Kalaskar M.G. (2019). Metal nanoparticles synthesis: An overview on methods of preparation, advantages and disadvantages, and applications. J. Drug Deliv. Sci. Technol..

[11] Wang K., Zhang Y.Q., Jiang L.C., Li Z.Y., Wang X., Zhai J.W., Zhang S.A. (2023). Understanding the effect of ambient gas pressure on the nanoparticle formation in electrically exploding wires. Phys. Plasmas.

[12] Suliz K., Miller A., Ivanov K., Pervikov A. (2022). Synthesizing multielement AlCrFeCuNi nanoparticles by joint electrical explosion of wires. Powder Technol..

[13] Pervikov A., Lozhkomoev A., Bakina O., Lerner M. (2019). Synthesis of core-shell and Janus-like nanoparticles by non-synchronous electrical explosion of two intertwined wires from immiscible metals. Solid State Sci..

[14] Pervikov A.V., Kazantsev S.O., Lozhkomoev A.S., Lerner M.I. (2020). Bimetallic Al–Ag, Al–Cu and Al–Zn nanoparticles with controllable phase compositions prepared by the electrical explosion of two wires. Powder Technol..

[15] Liang L.W., Wu J., Yin Z.K., Kong C.C., Pervikov A., Shi H.T., Li X.W., Qiu A.C. (2024). Synthesis of FCC structure Fe_10_Co_25_Ni_34_Cu_23_Al_8_ high-entropy-alloy nanoparticles by electrical wire explosion: For electromagnetic wave absorption. Appl. Phys. Lett..

[16] Caposciutti G., Tellini B., Saccomandi P., Cigada A. (2023). Experimental analysis on the exploding wire process for nanopowder production: Influence of initial energy and exploding atmosphere. Acta IMEKO.

[17] Pervikov A., Lerner M. (2017). Mechanism of the formation of the structure and phase state of binary metallic nanoparticles obtained by the electric explosion of two wires made of different metals. Curr. Appl. Phys..

[18] Sarkisov G.S., Sasorov P.V., Struve K.W., McDaniel D.H. (2004). State of the metal core in nanosecond exploding wires and related phenomena. J. Appl. Phys..

[19] Chen P.C., Liu X.L., Hedrick J.L., Xie Z., Wang S.Z., Lin Q.Y., Hersam M.C., Dravid V.P., Mirkin C.A. (2016). Polyelemental nanoparticle libraries. Science.

[20] Sarkisov G.S., Rosenthal S.E., Cochrane K.R., Struve K.W., Deeney C., McDaniel D.H. (2005). Nanosecond electrical explosion of thin aluminum wires in a vacuum: Experimental and computational investigations. Phys. Rev. E.

[21] Shi Z.Q., Shi Y.J., Wang K., Jia S.L. (2016). Experimental investigation on the energy deposition and morphology of the electrical explosion of copper wire in vacuum. Phys. Plasmas.

[22] Dinnebier R.E., Billinge S.J.L. (2008). Powder Diffraction: Theory and Practice.

[23] Baksht R.B., Tkachenko S.I., Romanova V.M., Mingaleev A.R., Oreshkin V.I., Ter-Oganes’yan A.E., Khattatov T.A., Shelkovenko T.A., Pikuz S.A. (2013). Stratification dynamics and the development of electrothermal instability at the wire explosion. Tech. Phys..

[24] Campbell F.C. (2008). Elements of Metallurgy and Engineering Alloys.

[25] Baker H., Okamoto H., Henry S.D., Davidson G.M., Fleming M.A., Kacprzak L., Lampman H.F. (1992). ASM Handbook Volume 3: Alloy Phase Diagrams.

[26] Coury F.G., Butler T., Chaput K., Saville A., Copley J., Foltz J., Mason P., Clarke K., Kaufman M., Clarke A. (2018). Phase equilibria, mechanical properties and design of quaternary refractory high entropy alloys. Mater. Des..

[27] Liu Q.L., Luo N., Fu Z.W., Niu B.B., Wu X.C., Wang X.B., Mei D., Li Q.K., Song B., He J.L. (2025). Additive manufacturing of immiscible alloys: Breaking the preparation bottleneck and unlocking the property potential. J. Alloys Compd..

[28] Chang X.J., Zeng M.Q., Liu K.L., Fu L. (2020). Phase engineering of high-entropy alloys. Adv. Mater..

[29] Oreshkin V.I., Baksht R.B. (2020). Wire Explosion in Vacuum. IEEE Trans. Plasma Sci..

[30] Zhang Q., Xu H., Tan X.H., Hou X.L., Wu S.W., Tan G.S., Yu L.Y. (2017). The effects of phase constitution on magnetic and mechanical properties of FeCoNi(CuAl)_x_ (x = 0–1.2) high-entropy alloys. J. Alloys Compd..

[31] Tian F.Y., Delczeg L., Chen N.X., Varga L.K., Shen J., Vitos L. (2013). Structural stability of NiCoFeCrAl_x_ high-entropy alloy from ab initio theory. Phys. Rev. B.

[32] Guo S., Ng C., Lu J., Liu C.T. (2011). Effect of valence electron concentration on stability of fcc or bcc phase in high entropy alloys. J. Appl. Phys..

[33] Chen R.R., Qin G., Zheng H.T., Wang L., Su Y.Q., Chiu Y., Ding H.S., Guo J.J., Fu H.Z. (2018). Composition design of high entropy alloys using the valence electron concentration to balance strength and ductility. Acta Mater..

[34] Zhang Y., Zuo T.T., Tang Z., Gao M.C., Dahmen K.A., Liaw P.K., Lu Z.P. (2014). Microstructures and properties of high-entropy alloys. Prog. Mater. Sci..

[35] Wang K., Zhang S.A., Li Z.Y., Wang T.Y., Wang J.C., Xu Y.B. (2025). Understanding the phase formation of binary nanoparticles in electrically exploding twin-wires. Phys. Plasmas.

[36] Lee Y.T., More R.M. (1984). An electron conductivity model for dense plasmas. Phys. Fluids.

[37] Desjarlais M.P. (2001). Practical improvements to the Lee-More conductivity near the metal-insulator transition. Contrib. Plasma Phys..

[38] Zhou X.W., Wadley H.N.G., Johnson R.A., Larson D.J., Tabat N., Cerezo A., Petford-Long A.K., Smith G.D.W., Clifton P.H., Martens R.L. (2001). Atomic scale structure of sputtered metal multilayers. Acta Mater..

[39] Zhou X.W., Johnson R.A., Wadley H.N.G. (2004). Misfit-energy-increasing dislocations in vapor-deposited CoFe/NiFe multilayers. Appl. Phys. Lett..

[40] Gafner Y.Y., Gafner S.L., Ryzkova D.A., Nomoev A.V. (2021). The role of gold atom concentration in the formation of Cu–Au nanoparticles from the gas phase. Beilstein J. Nanotechnol..

[41] Kumar L.S., Chakravarthy S.R., Verma R., Jayaganthan R., Sarathi R., Srinivasan A. (2020). Synthesis of multiphase binary eutectic Al-Mg alloy-nanoparticles by electrical wire explosion technique for high-energy applications, its characterisation and size-dependent thermodynamic and kinetic study. J. Alloys Compd..

[42] Yu P.F., Cheng H., Zhang L.J., Zhang H., Ma M.Z., Li G., Liaw P.K., Liu R.P. (2016). Nanotwin’s formation and growth in an Al-CoCuFeNi high-entropy alloy. Scr. Mater..

